# Supplementary material for: Monocyte-derived peritoneal macrophages protect C57BL/6 mice against surgery-induced adhesions
Source: Front Immunol. 2022 Oct 6;13:1000491. doi: 10.3389/fimmu.2022.1000491 (PMC9583908; doi:10.3389/fimmu.2022.1000491)
Supplement: Supplementary file 1 [file Presentation_1.pdf]

Supplementary Table 1. Adhesion scoring system based on the type of adhesion

		**Injured peritoneum**		**Incision site**	
	No adhesion	Caecum to injured peritoneum	Pelvic fat/omentum to injured peritoneum/caecum	Pelvic fat/omentum to incision site	Organ(s) to incision site/injury site
Score	0	1	2	3	4

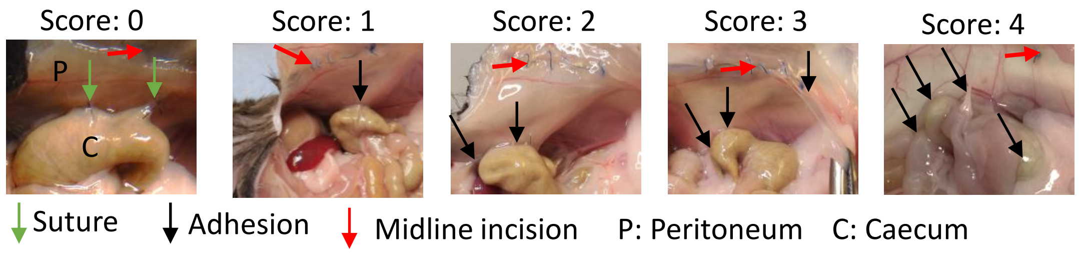

Supplementary Table 2. Panel 1 flow cytometry

Filter	Fluorochrome	Antigen	Clone	Dilution	Supplier
355 450_50	BUV440	Live/Dead	Zombie UV	1:1000	Biolegend
355 735_30	BUV737	Ym1 biotin	BAF2446	1:83	R& D System
405 450_50	PB	CD19	6D5	1:300	Biolegend
405 450_50	BV421	SigF	S17007L	1:300	Biolegend
405 450_50	PB	TCRβ	H57-597	1:300	Biolegend
405 450_50	PB	Ly6G	1A8	1:300	Biolegend
405 450_50	BV421	NK1.1	PK136	1:300	Biolegend
405 450_50	BV421	Ter119	TER-119	1:300	Biolegend
405 525_50	BV510	Ly6C	HK1.4	1:300	Biolegend
405 605_40	BV605	CD11c	N418	1:300	Biolegend
405 677_20	BV650	CD102	3C4(mIC2/4)	1:300	BD Biosciences
405 710_50	BV711	CD11b	M1/70	1:500	Biolegend
405 750_30	BV750-P	TNFa	MP6-XT22	1:166	Biolegend
405 810_40	BV786	Sca-1	D7	1:500	Biolegend
488 530_30	AF488	Relm-a	500-P214-100	1:166	Peprotech
488 710_50	PerCPeF 710	CD73	eBioTY/11.8	1:833	Invitrogen
561 586_15	PE	GATA6	D61E4	1:166	Cell Signaling Technology
561 610_20	PE-CF594	F4/80	BM8	1:300	Biolegend
561 670_30	PE-Cy5	CD45	30-F11	1:666	Biolegend
561 780_60	PE-Cy7	Tim4	RMT4-54	1:833	Biolegend
637 670_30	AF647	CD115	AFS98	1:300	Biolegend
637 730_45	AF700	I-A/I-E	M5/114.15.2	1:833	Biolegend
637 780_60	APC-cy7	CD226	10E5	1:300	Biolegend

Supplementary Table 3. Panel 2 flow cytometry

Filter	Fluorochrome	Antigen	Clone	Dilution	Suplier
355 450_50	BUV440	Live/Dead	Zombie UV	1:1000	Biolegend
355 735_30	BUV737	CD206 biotin	C068C2	1:166	Biolegend
405 450_50	PB	CD19	6D5	1:300	Biolegend
405 450_50	BV421	SigF	S17007L	1:300	Biolegend
405 450_50	PB	TCRβ	H57-597	1:300	Biolegend
405 450_50	PB	Ly6G	1A8	1:300	Biolegend
405 450_50	BV421	NK1.1	PK136	1:300	Biolegend
405 450_50	BV421	Ter119	TER-119	1:300	Biolegend
405 525_50	BV510	Ly6C	HK1.4	1:300	Biolegend
405 605_40	BV605	CD11c	N418	1:300	Biolegend
405 677_20	BV650	CD102	3C4(mIC2/4)	1:300	BD Biosciences
405 710_50	BV711	CD11b	M1/70	1:500	Biolegend
405 810_40	BV786	Ki76	B56	1:50	BD Biosciences
488 530_30	AF488	Relma	500-P214-100	1:166	Peprotech
488 710_50	PerCPeF 710	CD73	eBioTY/11.8	1:833	Invitrogen
561 586_15	PE	Lyve-1	10/FR2	1:166	Biolegend
561 610_20	PE-CF594	F4/80	BM8	1:300	Biolegend
561 670_30	PE-Cy5	CD45	30-F11	1:666	Biolegend
561 780_60	PE-Cy7	Tim4	RMT4-54	1:833	Biolegend
637 670_30	AF647	sema4a	5E3/SEMA4A	1:166	Biolegend
637 730_45	AF700	I-A/I-E	M5/114.15.2	1:833	Biolegend
637 780_60	APC-cy7	CD115	AFS98	1:300	Biolegend

Supplementary Table 4. The coating and detection antibody for ELISA

Cytokine	Coating antibody clone	Supplier	Detection antibody clone	Supplier
IL-4	11B11	Biolegend	BVD6-24G2	Biolegend
IL-10	JES5-16E3	Biolegend	JES5-2A5	Biolegend
IL-13	eBio13A	Invitrogen	eBio1316H	Invitrogen
Relm-α	500-P214	Peprotech	500-P214Bt	Peprotech
Ym1	Duoset	R&D System	Duoset	R&D System
IFN-γ	Cat. 551216	BD Biosciences	XMG1.2	Biolegend
IL-12p40	C15.6	Biolegend	C17.8	Biolegend
IL-6	Cat. 554400	BD Biosciences	MP5-3C11	Biolegend
TNF-α	MP6-XT22	Biolegend	MP6-XT22	Biolegend

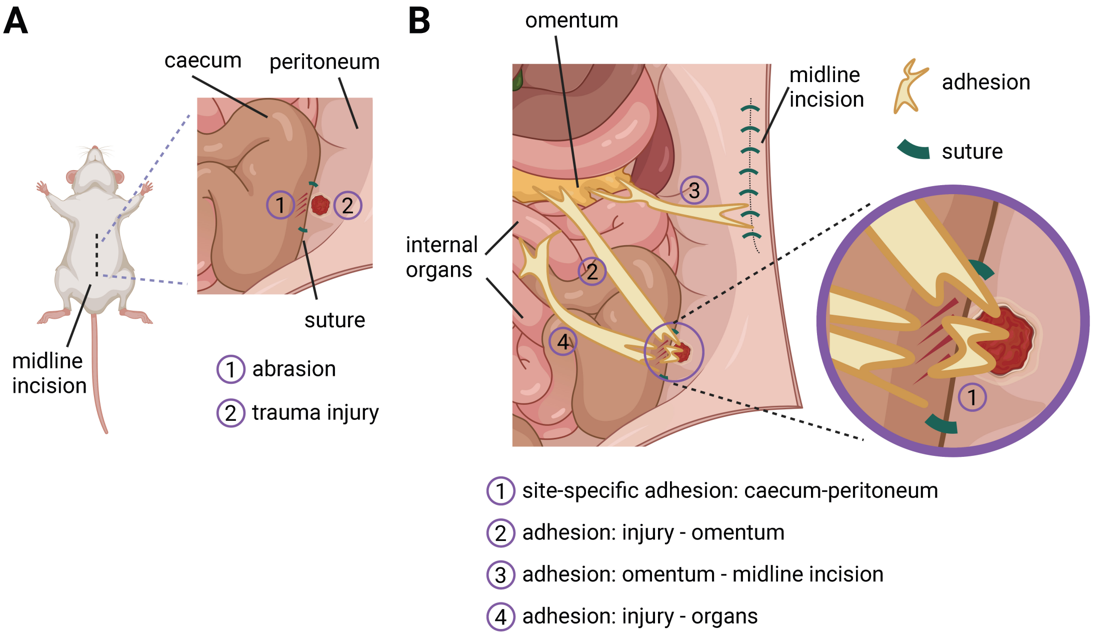

C

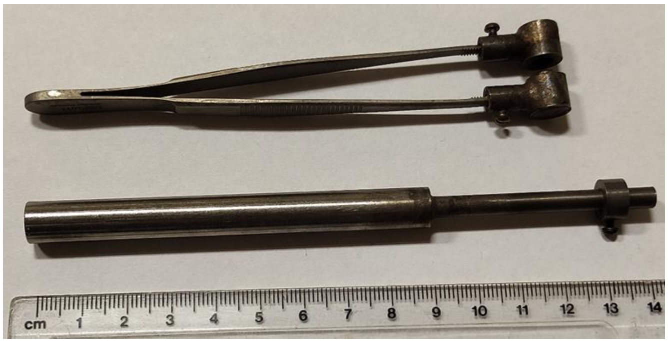

Supplementary Figure 1. Graphical illustration (A and B) of the experimental approach and image (C) of the trauma instrument used to induce peritoneal injury. The diagrams in A and B were created with BioRender.com.

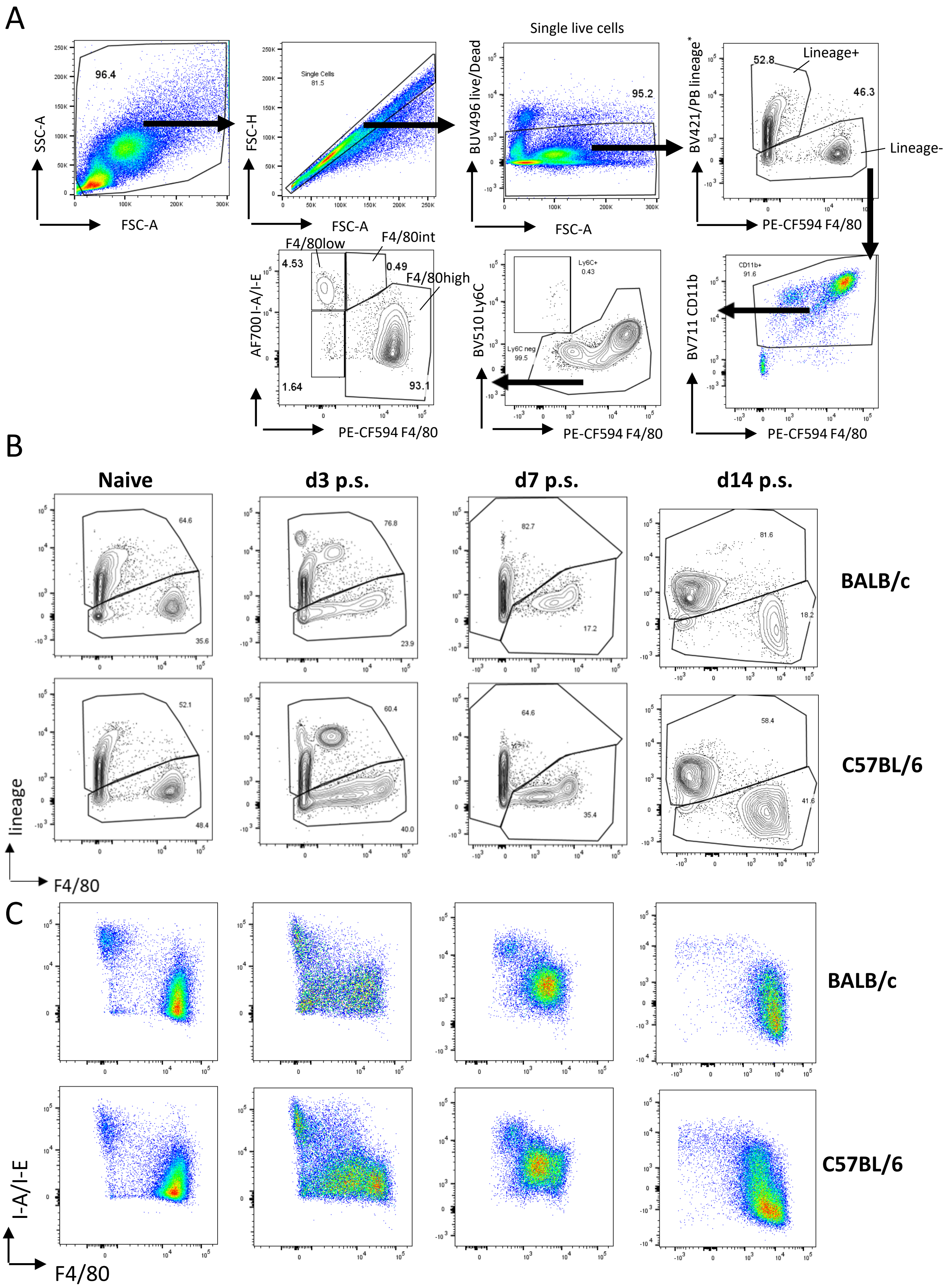

Supplementary Figure 2. (A) Gating strategy for flow cytometry analysis and representative gating of lineage+ or lineage- cells followed by the identification of F4/80 high, F4/80 int and F4/80 low macrophages. **(B & C)** Exemplary plots of F4/80 against lineage (B) or I-A/I-E (C) in BALB/c and C57BL/6 prior at different timepoints after surgery. Lineage* (CD19, SigF, TCRb, Ly6G, NK1.1, and Ter119)

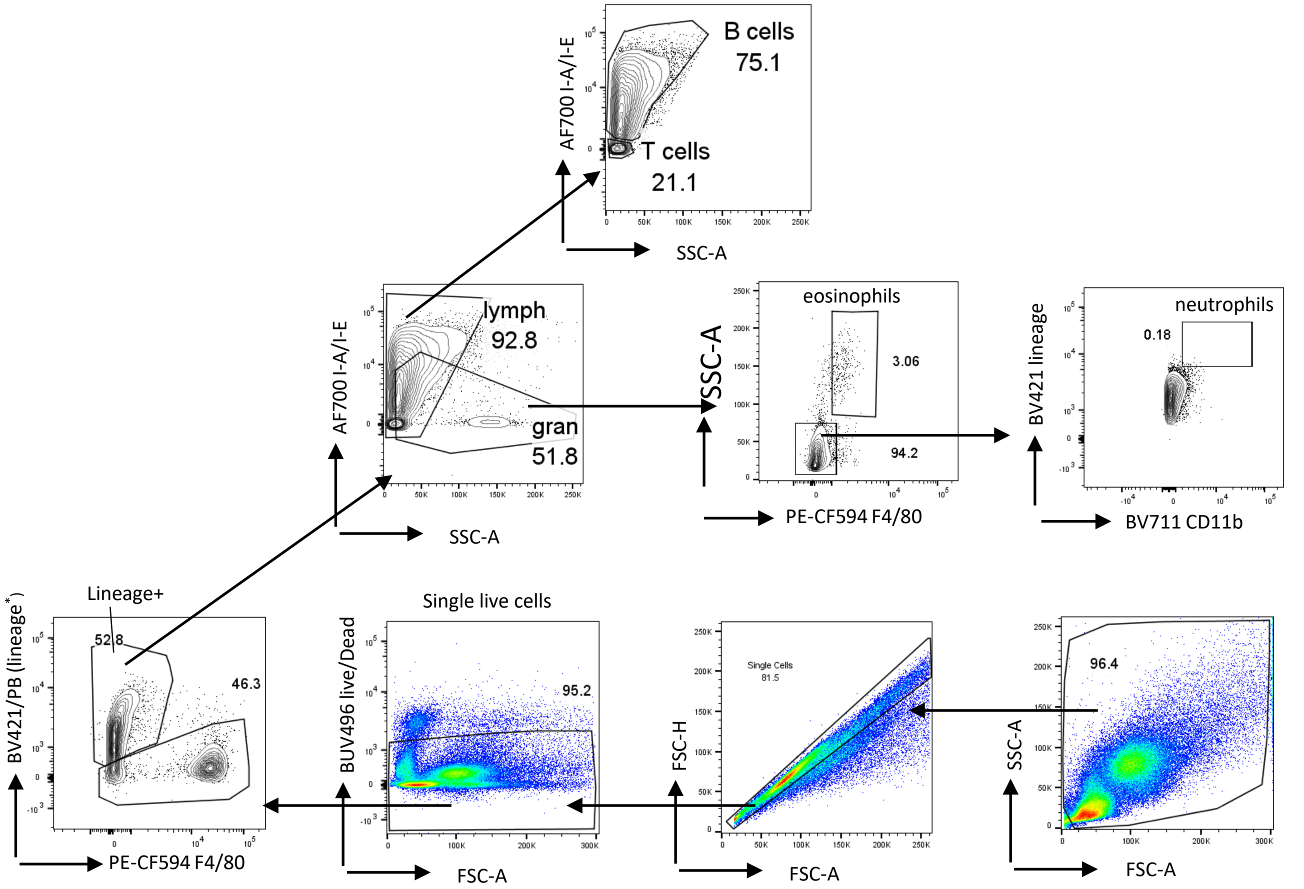

Supplementary Figure 3. Gating strategy for flow cytometry analysis of lineage+ populations. Lineage* (CD19, SigF, TCRb, Ly6G, NK1.1, and Ter119). lymph: lymphocytes; gran: granulocytes

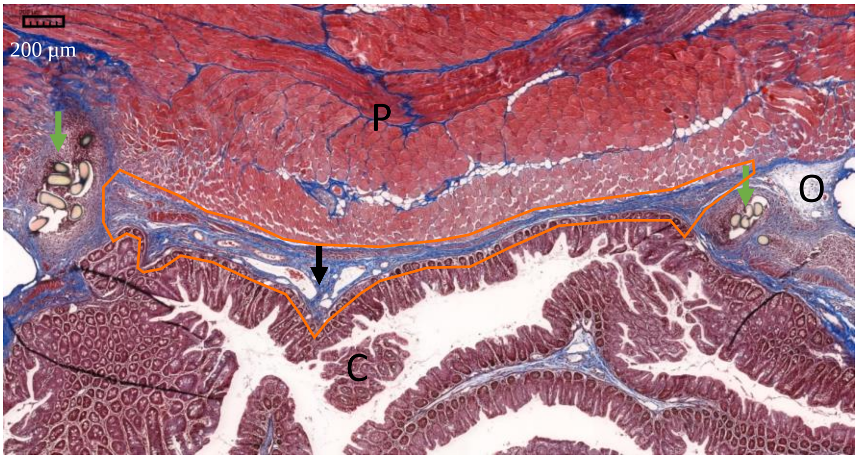

Suture Adhesion P: Peritoneum C: Caecum O: Omentum

orange line: site-specific adhesion area

Supplementary Figure 4. Exemplary illustration on how total collagen on site-specific adhesion area was measured. Total collagen within the site specific adhesion area was measured using RGB thresholding by QuantCentre and HistoQuant plugin on SlideViewer software (version 2.5). Scale bar: 200µm

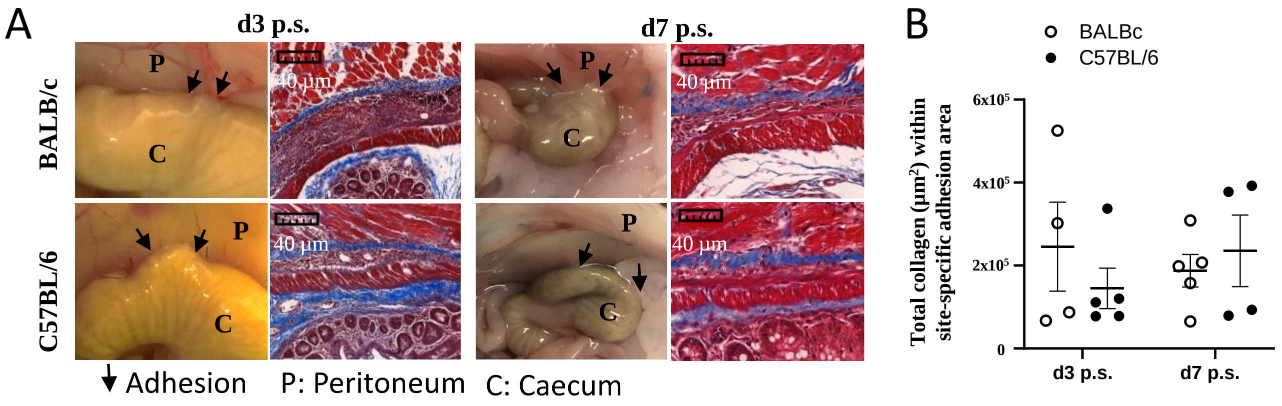

Supplementary Figure 5. (A) Representative photographs of adhesions and micrographs of Masson's trichrome stained histological sections at day 3 and 7 after surgery in BALB/c and C57BL/6 mice. Scale bar: 40µm. **(B)** Total collagen content in site-specific adhesions.

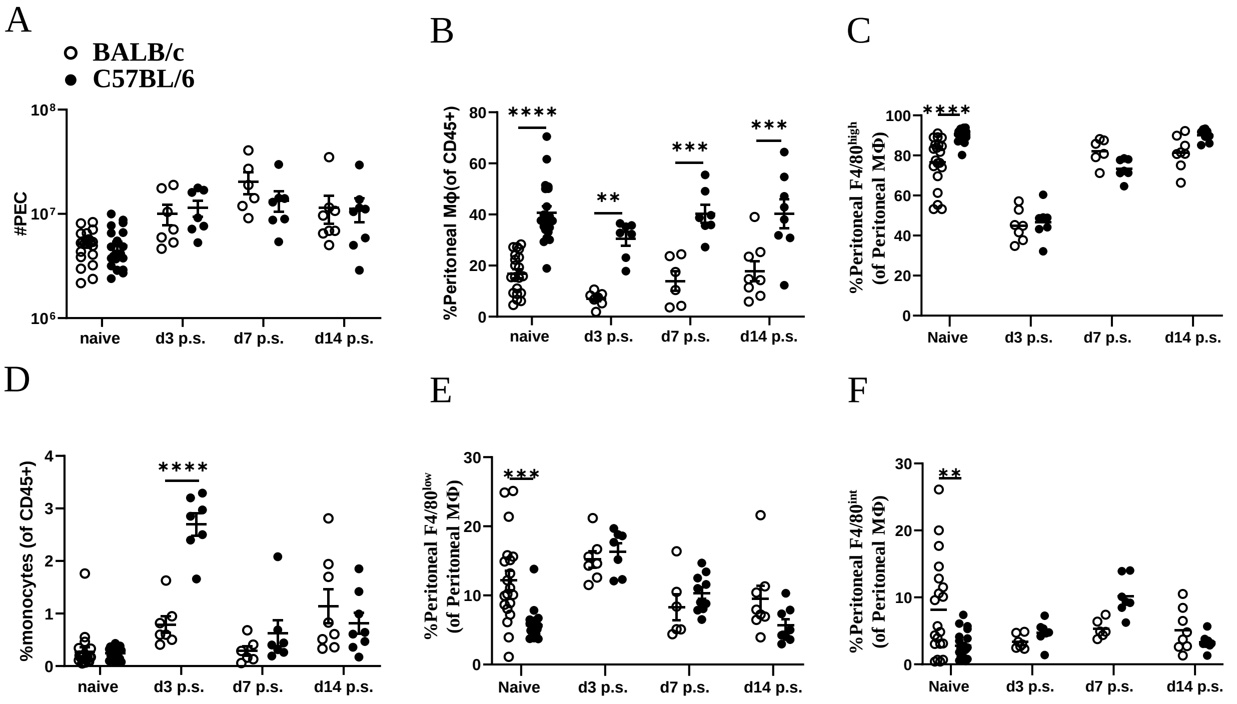

Supplementary Figure 6. Total Peritoneal exudate cells and relative percentage of peritoneal macrophages and monocytes in naïve mice or at various timepoints after surgery.

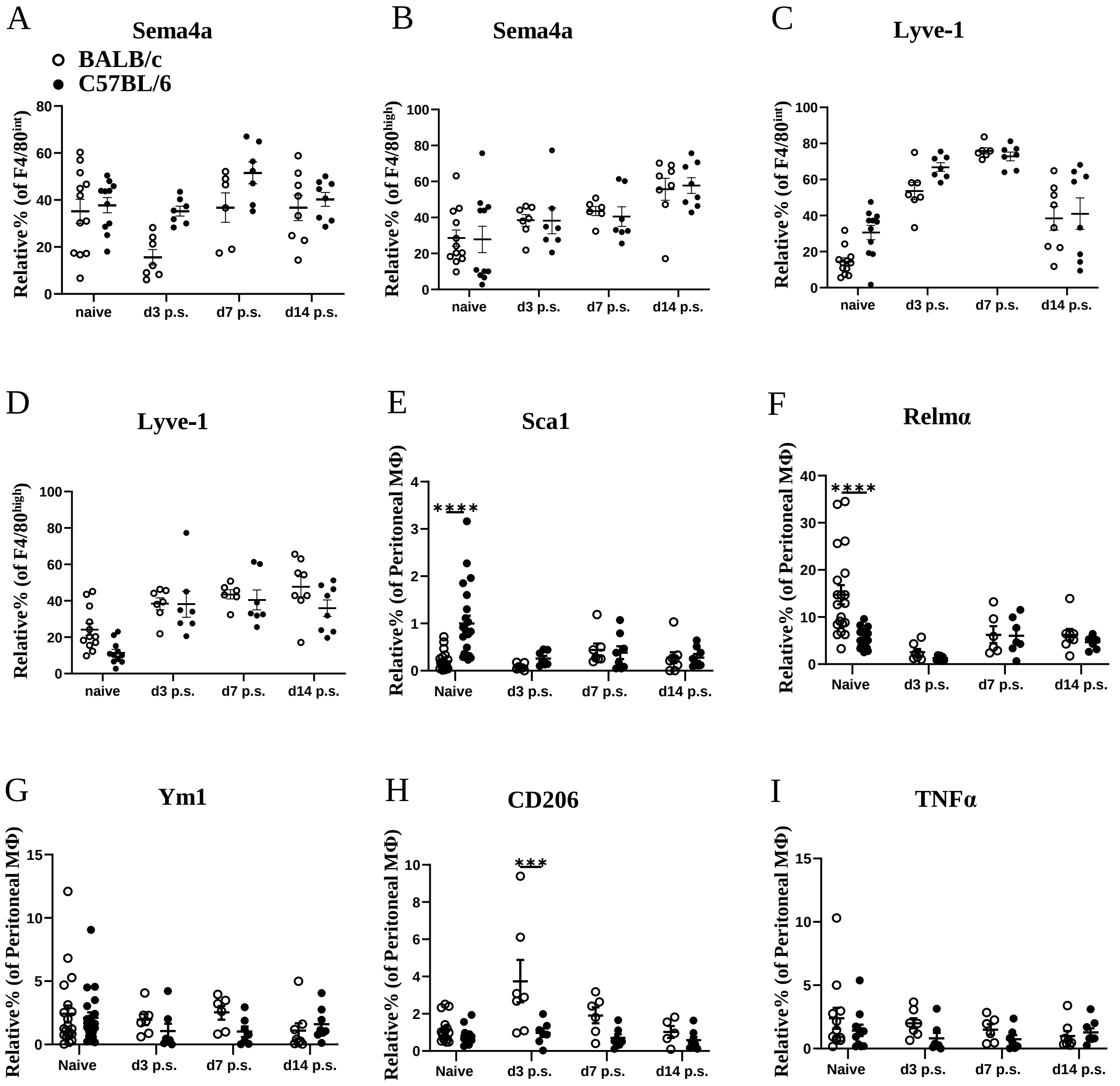

Supplementary Figure 7. Relative percentages for different markers expressed by peritoneal macrophages within BALB/c and C57BL/6 mice prior and post-surgery.

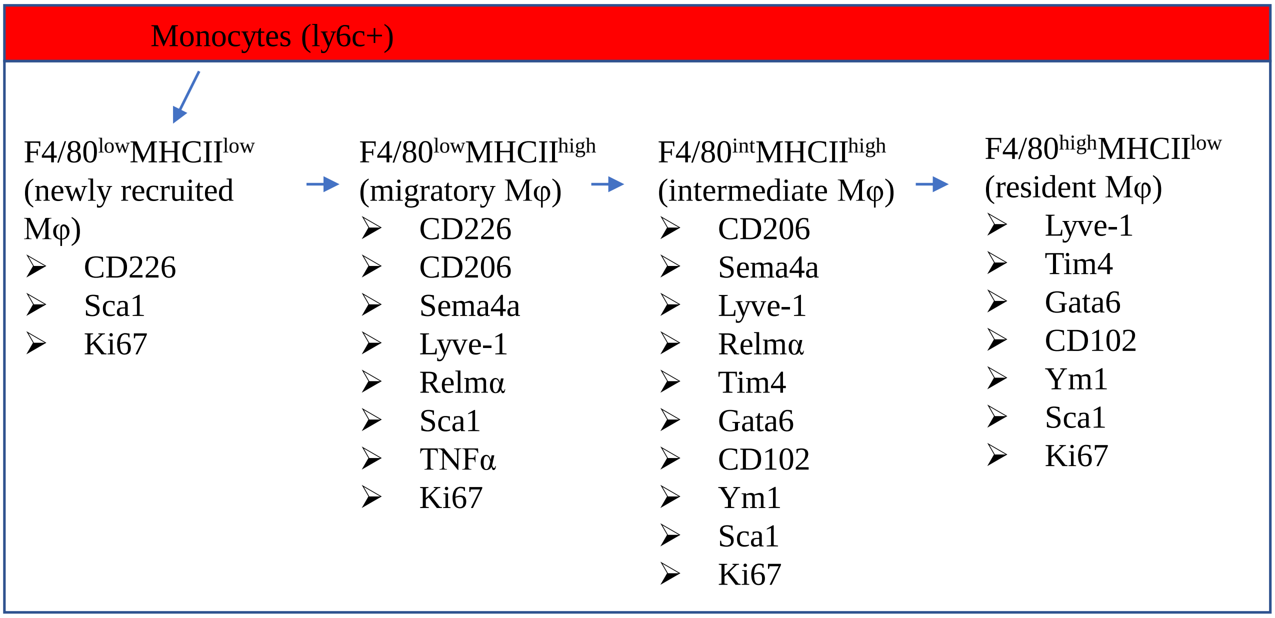

Supplementary Figure 8. Schematic representation of how different markers of peritoneal macrophages were expressed in different subsets

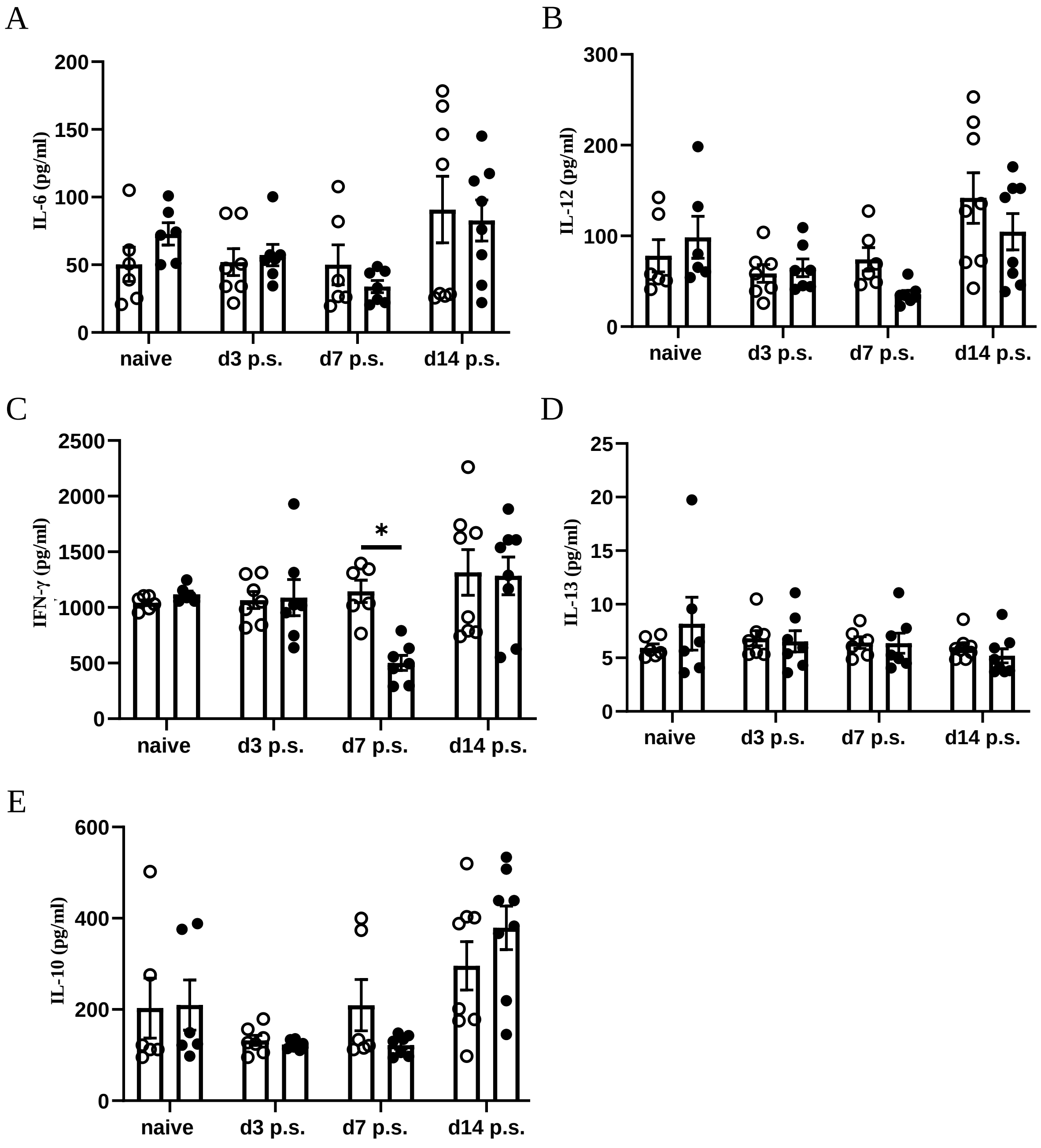

Supplementary Figure 9. Cytokine analysis in the peritoneal lavage of BALB/c and C57BL/6 before and after surgery.

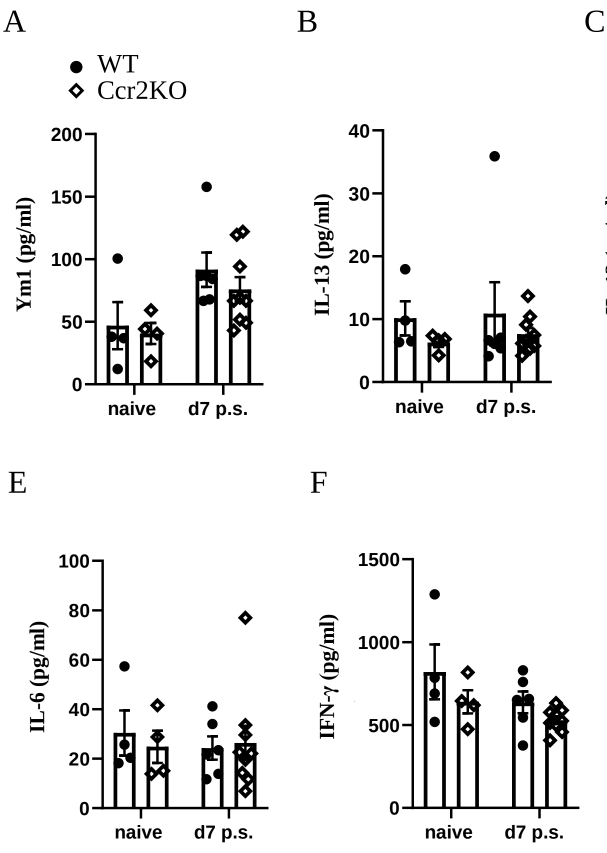

Supplementary Figure 10. Cytokine analysis in the peritoneal lavage of Ccr2KO and WT littermate before and after surgery.

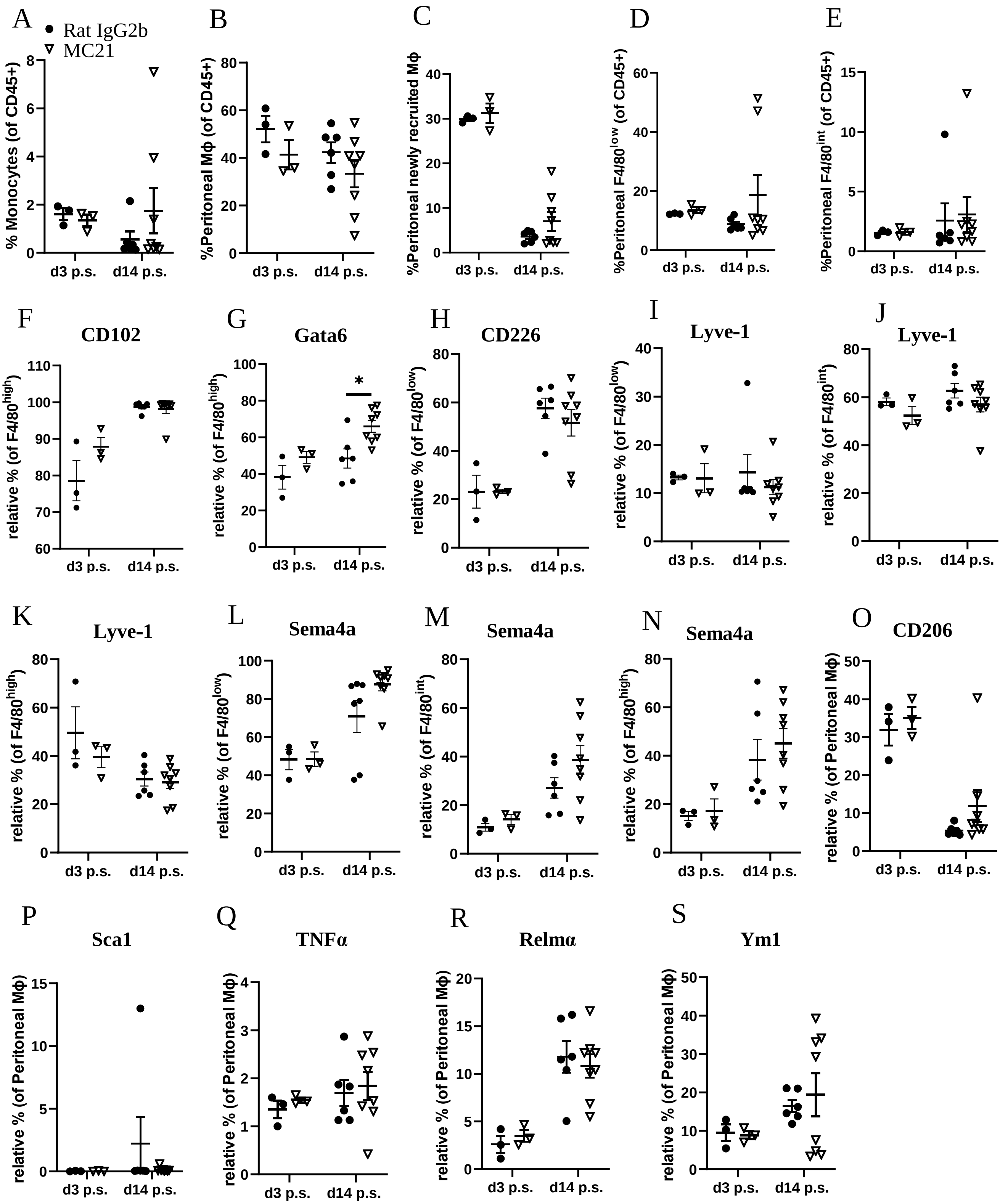

Supplementary Figure 11. Relative percentage of monocytes and different markers of peritoneal macrophages of Rat IgG2b isotype control and anti-CCR2 (clone MC21) treated mice after surgery. Peritoneal newly recruited macrophages are F4/80^{low}MHCII^{low}.

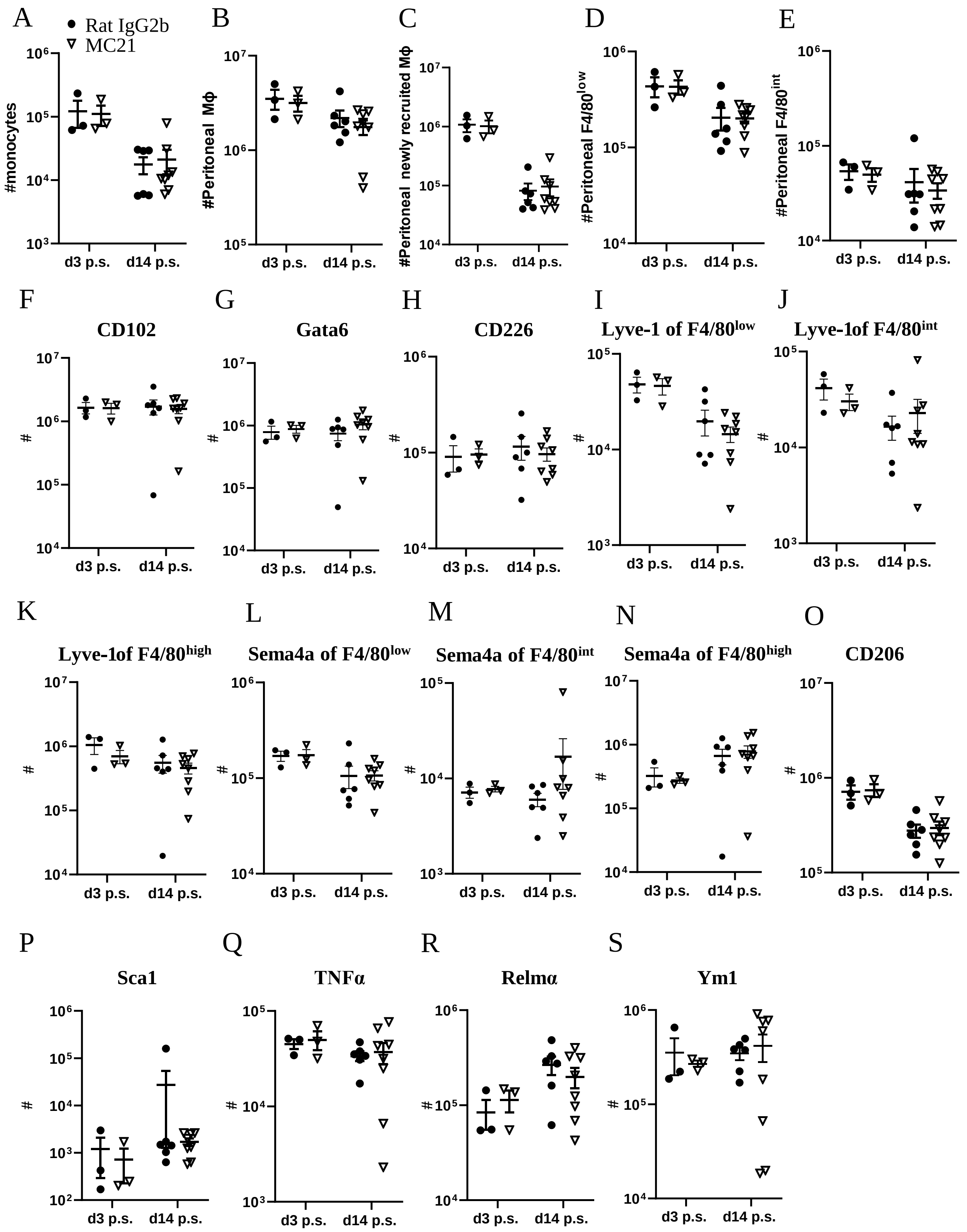

Supplementary Figure 12. Total cell number of monocytes and different markers of peritoneal macrophages of Rat IgG2b isotype control and anti-CCR2 (clone MC21) antibody treated mice after surgery. Peritoneal newly recruited macrophages are F4/80^{low}MHCII^{low}.

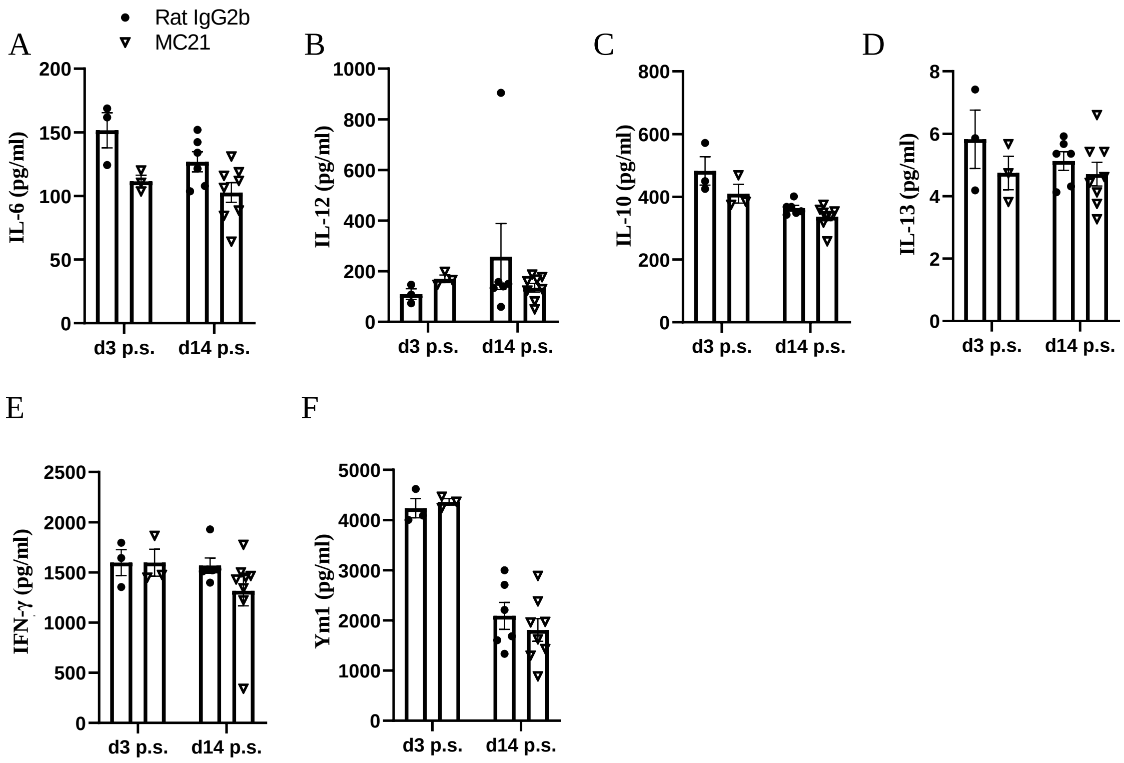

Supplementary Figure 13. Cytokine analysis in the peritoneal lavage of Rat IgG2b isotype control and anti-CCR2 (clone MC21) antibody treated mice after surgery.

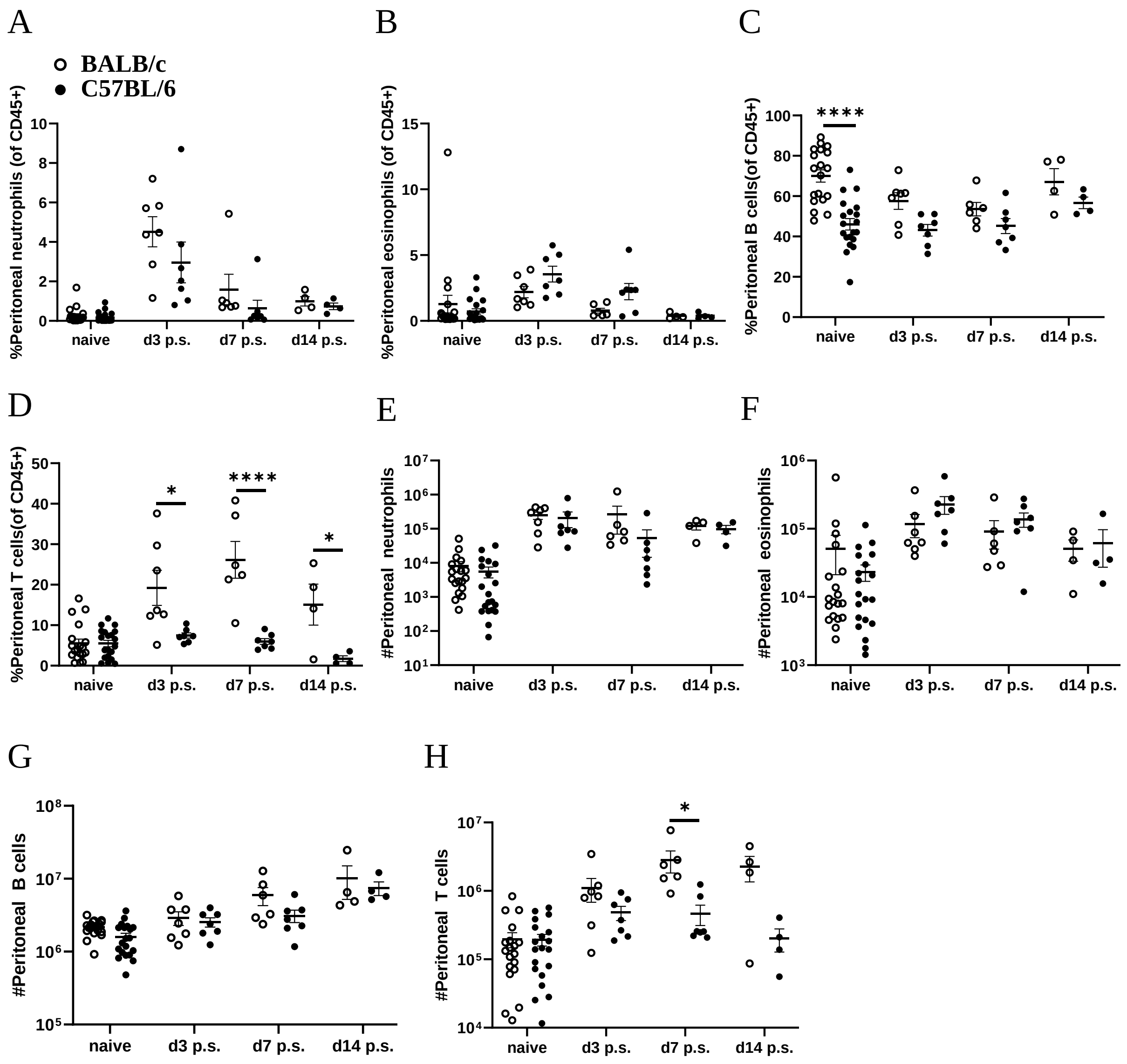

Supplementary Figure 14. The relative percentage and total cell number of neutrophils, eosinophils, B cells, and T cells in the peritoneal cavity of BALB/c and C57BL/6 mice prior and after surgery.
